# A Metabolomics Approach to the Identification of Urinary Biomarkers of Pea Intake

**DOI:** 10.3390/nu10121911

**Published:** 2018-12-04

**Authors:** Pedapati S.C. Sri Harsha, Roshaida Abdul Wahab, Catalina Cuparencu, Lars Ove Dragsted, Lorraine Brennan

**Affiliations:** 1 School of Agriculture and Food Science, Institute of Food and Health, University College Dublin, Belfield, Dublin 4, Ireland; siva.pedapati@ucd.ie (P.S.C.S.H.); roshaida.abdul-wahab@ucdconnect.ie (R.A.W.); 2 Department of Nutrition, Exercise and Sports, Faculty of Science, University of Copenhagen, 1958 Frederiksberg C, Denmark; cup@nexs.ku.dk (C.C.); ldra@nexs.ku.dk (L.O.D.)

**Keywords:** metabolomics, biomarkers, dietary assessment, peas

## Abstract

A significant body of evidence demonstrates that isoflavone metabolites are good markers of soy intake, while research is lacking on specific markers of other leguminous sources such as peas. In this context, the objective of our current study was to identify biomarkers of pea intake using an untargeted metabolomics approach. A randomized cross-over acute intervention study was conducted on eleven participants who consumed peas and couscous (control food) in random order. The urine samples were collected in fasting state and postprandially at regular intervals and were further analysed by ultra-performance liquid chromatography coupled to quadrupole time of flight mass spectrometry (UPLC-QTOF-MS). Multivariate statistical analysis resulted in robust Partial least squares Discriminant Analysis (PLS-DA) models obtained for comparison of fasting against the postprandial time points (0 h vs. 4 h, (R^2^X = 0.41, Q^2^ = 0.4); 0 h vs. 6 h, ((R^2^X = 0.517, Q^2^ = 0.495)). Variables with variable importance of projection (VIP) scores ≥1.5 obtained from the PLS-DA plot were considered discriminant between the two time points. Repeated measures analysis of variance (ANOVA) was performed to identify features with a significant time effect. Assessment of the time course profile revealed that ten features displayed a differential time course following peas consumption compared to the control food. The interesting features were tentatively identified using accurate mass data and confirmed by tandem mass spectrometry (MS using commercial spectral databases and authentic standards. 2-Isopropylmalic acid, asparaginyl valine and N-carbamoyl-2-amino-2-(4-hydroxyphenyl) acetic acid were identified as markers reflecting pea intake. The three markers also increased in a dose-dependent manner in a randomized intervention study and were further confirmed in an independent intervention study. Overall, key validation criteria were met for the successfully identified pea biomarkers. Future work will examine their use in nutritional epidemiology studies.

## 1. Introduction

Diet is one of the major lifestyle related risks associated with a wide range of chronic diseases [1,2,3]. However, the exact relationship between diet components and health still needs to be determined and it is a prerequisite to achieve accurate measurement of food intake. Traditional dietary assessment methods include self-reporting platforms such as food frequency questionnaires (FFQ), 24 h dietary recalls and food diaries. These self-reported tools have a number of well documented limitations such as energy underreporting, recall errors and misjudgement in the estimation of portion sizes and therefore attenuate the diet and disease relationship [4,5,6]. To overcome some of these issues, food intake biomarkers have emerged as a more objective measurement and could be an adjunct to the existing assessment methods [7,8,9]. These biomarkers measure in human biofluids reflect food intake [10]. The emergence of metabolomics technology has resulted in the discovery of putative biomarkers of exposure to a range of foods and food groups such as cruciferous vegetables [11,12], citrus fruits [13,14,15], red meat [16], sugar-sweetened beverages [17], coffee [18], and black tea [19]. Some of the well-established and validated food intake biomarkers include urinary tartaric acid for determination of red grape intake [20], urinary proline-betaine for citrus fruit consumption [21] and alkylrescorinols [22]. However, we still lack validated biomarkers for a range of foods that are important for health and legumes are an example.

Legumes are fruits or seeds of a plant belonging to the Fabaceae family and are consumed as traditional diets in many regions of the world. Well known legumes include peas, beans, lentils, lupins, soybeans and peanuts. Legumes are rich sources of vegetable protein and provide slow, digestible, complex carbohydrates, soluble and insoluble fibres, various phytochemicals and micronutrients including iron, copper and manganese [23,24]. Legumes are associated with potential health benefits with evidence stemming from epidemiological studies [25,26,27,28]. Our previous work employing a systematic review of the literature reveals that to date the field of biomarkers of legume intake has focused predominantly on soy intake [29]. The isoflavone metabolites genistein and daidzein are reported as good markers of soy consumption [30,31]. On the other hand, there are very limited studies that have explored biomarkers of other leguminous sources such as pulses. For dry bean intake, serum markers such as pipecolic acid, *S*-methyl cysteine [32] and urinary kaempferol have been reported [33]. N-methylnicotinic acid was proposed as a biomarker related to consumption of peas, although it is reported as a non-specific marker due to its presence in higher amounts post consumption of other sources such as coffee, tea, cacao, etc. [34]. Although several health benefits [35] have been linked to pea intake, information on specific biomarkers is lacking. The objective of this study was to use a metabolomics-based approach to identify potential urinary biomarkers associated with pea intake.

## 2. Materials and Methods 

### 2.1. Acute Study: Ethical Approval, Subject Recruitment and Study Design

The present work was performed under the umbrella of the FOODBALL project [36] and the focus was biomarkers of legume intake. The acute study was approved by the Human Research Ethics Committee at University College Dublin (UCD) (LS-15-62-Brennan) and registered as a Clinical Trail (ISRCTN70680593). We aimed to recruit a total of 12 participants as previous studies had demonstrated that a samples size of 8–12 was sufficient to use metabolomics to identify biomarkers of food intake [37,38,39]. All procedures were conducted according to the principles expressed in the Declaration of Helsinki. Inclusion criteria were healthy men and women aged between 18 and 40 years; Caucasian; Body Mass Index (BMI) >18.5 and <30 kg/m^2^; non-smokers; not taking any medication (with the exception of oral contraceptive pill); not having any known or chronic infectious diseases; not planning a pregnancy or pregnant or lactating; and not on supplements or antibiotics. The recruitment was conducted via posters, college website advertisement, and word of mouth. The individuals who made contact were screened for eligibility and anthropometric measurements were taken. Body height was measured using the Leicester portable height measure (Chasmores), weight was measured using a Tanita body composition analyser BC-420MA (Tanita Corporation, Middlesex, UK) and waist and hip circumferences were measured using a non-stretch tape measure (Seca, Birmingham, UK). Height and weight were used to calculate BMI (weight (kg)/height (m^2^)), and waist and hip circumferences were used to calculate waist-hip ratio (waist (cm):hip (cm)). After successful completion of screening, the participants provided an informed consent form. All eligible participants were randomized to consume test food (peas, 138 g) and control food (couscous, 138 g) in a random order. 

Participants were asked to refrain from consuming alcohol and fruits and vegetables for 2 days prior to the study visit. On the day prior to the study visit, participants were asked to consume a standardized dinner (Chicken and rice meal) and to fast for 12 h prior to the appointment. On the morning of the study visit, participants collected a fasting first-void urine sample, which was immediately placed on ice in the urine kit bag provided and transported to the study centre. Following test/control food consumption, the urine samples were collected over the following intervals: 0–1, 1–2, 2–4, 4–6, 6–12, 12–24 and 48 h post consumption. Participants refrained from eating but were allowed to drink ~100 mL of water (provided every hour) for the first 6 h post consumption of the food. All urine samples were centrifuged at 1800× *g* for 10 min at 4 °C and 1-mL aliquots were stored at – 80 °C for further analysis. 

### 2.2. Dose–Response Study for Biomarker Validation

The dose–response study was approved by the Human Research Ethics Committee at University College Dublin (UCD) (LS-16-92-Brennan). All procedures were conducted according to the principles expressed in the Declaration of Helsinki. All the inclusion, recruitment and screening criteria were the same as the acute study. Participants (*n* = 12) consumed known quantities of peas daily for a period of 4 days and collected a fasting urine sample on Day 5. The study was repeated over 2 weeks so that three different quantities (low (40 g), medium (75 g), and high (165 g)) of peas were consumed. The portions were chosen according to “A photographic atlas of food portion sizes” which is designed to help people describe amounts of food consumed and is widely used by dieticians and nutritionists across UK and Ireland. On Days 1, 2, 3, and 4 of each intervention week, the participants consumed peas as part of their evening meal after 18:00. They were also asked to photograph the meal pre- and post-consumption to confirm consumption of the peas. On Day 5, a first void urine sample and a fasting blood sample were collected. Samples were processed as described above. On Day 5, a 24 h dietary record was also collected. 

### 2.3. Independent Acute Study for Biomarker Confirmation

An independent acute study at university of Copenhagen aimed to identify biomarkers of meat intake utilizing peas as a control food and was used here to confirm the markers of pea consumption. This study was approved by the Regional Ethics Committee for the capital area in Denmark (registration number H-15020401) and is registered at ClinicalTrials.gov (NCT03305718). 

The study design in terms of recruitment, inclusion and exclusion criteria was similar to the above-described study for peas biomarker identification, as it was conducted as part of the FOODBALL project. Briefly, the study was a randomized controlled four-way cross-over meal trial (*n* = 10) aimed at discovering biomarkers for intake of meat. The test meals comprised three types of meat and an iso-caloric control, i.e., green pea protein and egg white (pea burger-patty) and the volunteers consumed the foods in a random order. Urine samples were collected at regular intervals of time until 48 h post-consumption.

### 2.4. Nontargeted Ultra-Performance Liquid Chromatography Coupled to Quadrupole Time of Flight Mass Spectrometry (UPLC-QTOF-MS) Metabolite Profiling Analysis

The solvents acetonitrile and formic acid as well as standards and internal standards were purchased from Sigma-Aldrich (St. Louis, MO, USA). Reagent water was ion exchanged and purified further by a Millipore unit to obtain an electrical resistance of below 18 MΩ. 

#### 2.4.1. Standards

A standard mixture of 18 biologically relevant metabolites belonging to different compound classes (arginine, taurine, 1-methyl-L-histidine, citrulline, creatinine, malic acid, methionine, citric acid, succinic acid, ketoglutaric acid, isoleucine, fumaric acid, leucine, methylmalonic acid, glutaric acid, adipic acid, hippuric acid, and pimelic acid) and a mixture of four internal standards (malic d3, methionine d3, succinic acid d4, and adipic acid d4) were prepared. Both mixtures were used for quality control assessment of the analytical platform. The stock concentration of the individual standards was prepared at 1000 µg mL^−1^. Both standard and the internal standard mix were prepared at 10 mg/L concentration. 

#### 2.4.2. Sample Preparation 

The standard and internal standard mix working stock solutions, 1-mL aliquots of pooled urine (QC) and test urine sample were thawed for 20 min and centrifuged at 3884× *g* (VWR, Galaxy ministar, Vienna, Austria). Samples were prepared by adding 100 µL of standard/pooled urine (QC)/test urine to 100 µL of internal standard mix. The mixture was vortexed for 15 s at 25 Hertz (VELP scientifica, Usmate, Italy) and centrifuged at 200× *g* for 5 min. The supernatant was then transferred to autosampler vials with 250 µL inserts. 

#### 2.4.3. Sample Analysis 

The samples were analysed by UPLC-QTOF-MS (Agilent Technologies, Santa Clara, CA, USA), which consisted of 1290 Infinity II LC system and an Agilent Jetstream (AJS) Electrospray ionization (ESI) source coupled to a 6545 QTOF mass spectrometer. The samples were analysed by using reverse phase chromatography (RP). Data acquisition was performed using the Masshunter acquisition B.06.00 software (Agilent Technologies). The sample batch run commenced with a standard test mix, followed by 4 QCs to achieve a stable instrument response. Test urine samples were then injected in a randomized order followed by two QCs and a standard test mix to check for the instrument performance and stability over the data acquisition course. The RP analysis was performed on a Zorbax Eclipse plus C18 RRHD (2.1 × 50mm, 1.8 µm) coupled to a UHPLC guard column Zorbax Eclipse plus C18 (2.1 × 5mm, 1.8Micron). The column was maintained at 30 °C throughout the analysis and the injection volume was 5 µL. A flow rate of 0.4 mL/min was used with a linear gradient of 0.1% formic acid in water (Eluent A) and 0.1% formic acid in acetonitrile/water (80:20) (Eluent B). The gradient profile was as follows: 1% B (0–1.5 min); 11% B (1.5–9 min); 25% B (9–15 min); 50% B (15–18 min); 99% B (18–18.05min); 99% B (18.05–21 min); 1% B (21–21.05 min); and 1% B (21.05–23 min). The mass spectrometer was operated in both positive and negative ionization mode with Jetstream electrospray ionization (ESI) source. The MS parameters were as follows: drying gas temperature, 325 °C; drying gas flow rate, 10 L/min; sheath gas temperature, 350 °C; sheath gas flow rate, 11 L/min; nebulizer pressure, 45 gauge pressure (pounds per square inch); capillary voltage, 3500 V; nozzle voltage, 1000 V; fragmentor voltage, 100 V; and skimmer, 45 V. A 2-GHz extended dynamic range mode was used and the accurate mass spectra was acquired over a mass range of *m*/*z* 50–1600. The data were collected in centroid mode at scan rate of 1 spectra/s and an abundance threshold of 300. The targeted MS/MS was performed with the same chromatographic and MS conditions as described above. The data were acquired at MS/MS scan range of 40–700 *m*/*z* at a scan rate of 1 spectra/s. Collision energies of 10 eV, 20 eV and 40 eV were applied.

For the independent acute study, the urine samples were analysed on a UPLC-ESI-QTOF/MS high-performance liquid chromatographic system (Acquity UPLC, QTOF Premier, Waters, Manchester, UK) as previously described [40]. 

### 2.5. Data Treatment and Analysis

The acquired data were processed using MassHunter Qualitative Analysis B.07.00 software (Agilent Technologies, Santa Clara, CA, USA). The molecular feature extractor (MFE) algorithm was used to extract molecular features characterized by retention time (RT) corresponding *m/z* values representing different adducts or isotopes of the same compound and provides details of the signal intensity and accurate mass. The target data type was set to small molecules and the threshold value for peak height was set to 5000 counts. The isotopic peak spacing tolerance was 0.0025 *m/z* with maximum charge state of 1 and isotope model was set to common organic molecules. The data output was then transferred to Compound Exchange Format (.cef) files. These .cef files were loaded on to Mass profiler software (Version B.07.01; Agilent technologies) used for compound alignment and data filtration. The alignment of RT and *m/z* values was set using a tolerance window of 0.3 min and 2.0 mDa, respectively. The reduction in the number of molecular features was enabled by setting the sample occurrence frequency by 50% across all samples. Missing values were replaced by half of the minimum value of that particular feature abundance (or corresponding variable). All feature abundance values were normalized to osmolality measured using an Advanced Micro Osmometer model 3D3 (Advanced Instruments, Norwood, MA, USA). For the independent acute study, the acquired data were pre-processed using mzMine, as described previously [40].

Multivariate statistical analysis was performed using Simca-P software (version 13.0.3, Umetrics, Sartorius Stedim Biotech, Umea, Sweden). Datasets were scaled using Pareto scaling. Principal component analysis (PCA) reduced the dataset to a small number of principal components. Partial least-squares discriminant analysis (PLS-DA) was used to identify the features that discriminated between the groups. PLS-DA models obtained were validated by permutation testing. The variable importance of projection (VIP) was used to identify the most influential variables with a cut-off of 1.5. Repeated measures ANOVA was performed to identify metabolites with a significant time effect. All *p* values were corrected for multiple comparisons (Metaboanalyst 3.0). The interesting features were identified using MassHunter ID browser (Version B.07.00, Agilent technologies) by molecular formula generation and an accurate mass data search using a personal compound database library obtained from METLIN public database. MS/MS was performed to attain structural confidence for the metabolites identified by the accurate mass. Each metabolite was attributed to different levels of identification as illustrated [41]. Briefly, four levels of confidence in metabolite identification were reported: Level I (identified compounds); Level II (putatively annotated compounds); Level III (putatively characterized compound classes); and Level IV (Unknowns). For MS/MS compound identification, sources such as authentic reference standards, competitive fragmentation modelling for metabolite identification (CFM-ID 2.0, http://cfmid.wishartlab.com) and molecular structure correlator (Version B.08.00, Agilent technologies) platforms were used.

## 3. Results

### 3.1. Discovery Study Characteristics

An overview of the recruitment process is reported in Figure S1. The details of study population characteristics are presented in Table 1. The average age was 25 ± 4.2 and the average BMI was 24 ± 2.9 kg/m^2^.

### 3.2. Identification of Features Related to Pea Consumption

**Figure 1:**
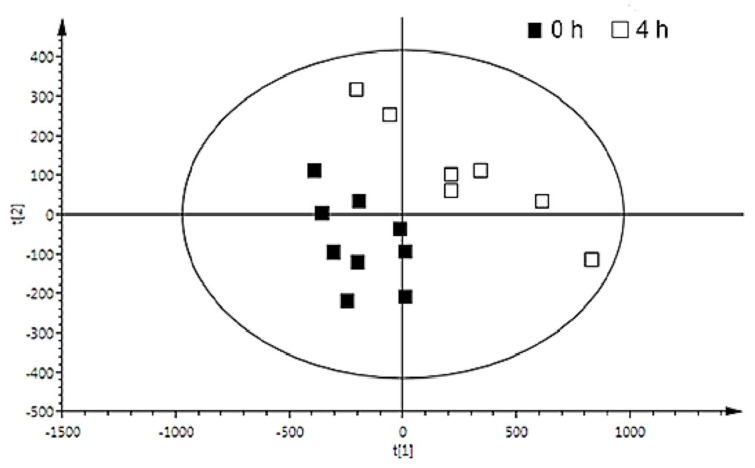
Partial least square discriminant analysis (PLS-DA) of Ultra-Performance Liquid Chromatography coupled to Quadrupole Time of Flight Mass Spectrometry (UPLC-QTOF-MS) urine data of time point 0 (■) and time point 4 h post consumption of peas (□). Q^2^, 0.4; R^2^X, 0.41; t [1], PLS component 1; t [2], PLS component 2. At the timepoint 4 h, urine samples were unavailable for two participants.

A total of 73,734 (positive mode) and 84,161 (negative mode) features were detected across all samples using molecular feature extractor. Further data processing and filtration steps by mass profiler resulted in reduction of features and a dataset comprising a total of 819 (+ve) and 1004 (−ve) features was obtained. PCA was applied to the dataset to explore trends in the data. An example of the PCA for the baseline and the time point 4 h post consumption of peas is depicted (Figure S2). Robust PLS-DA models revealed discrimination between baseline urinary profiles and post-pea consumption profiles. For example, baseline and the time point 4 h post-pea consumption model had a Q^2^ value of 0.4 for negative ion mode data (Figure 1). 

**Figure 2:**
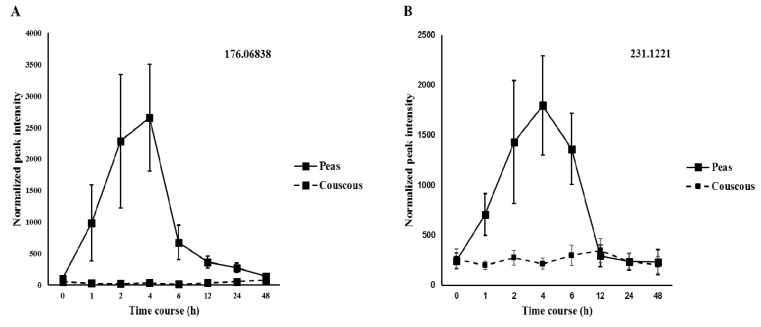

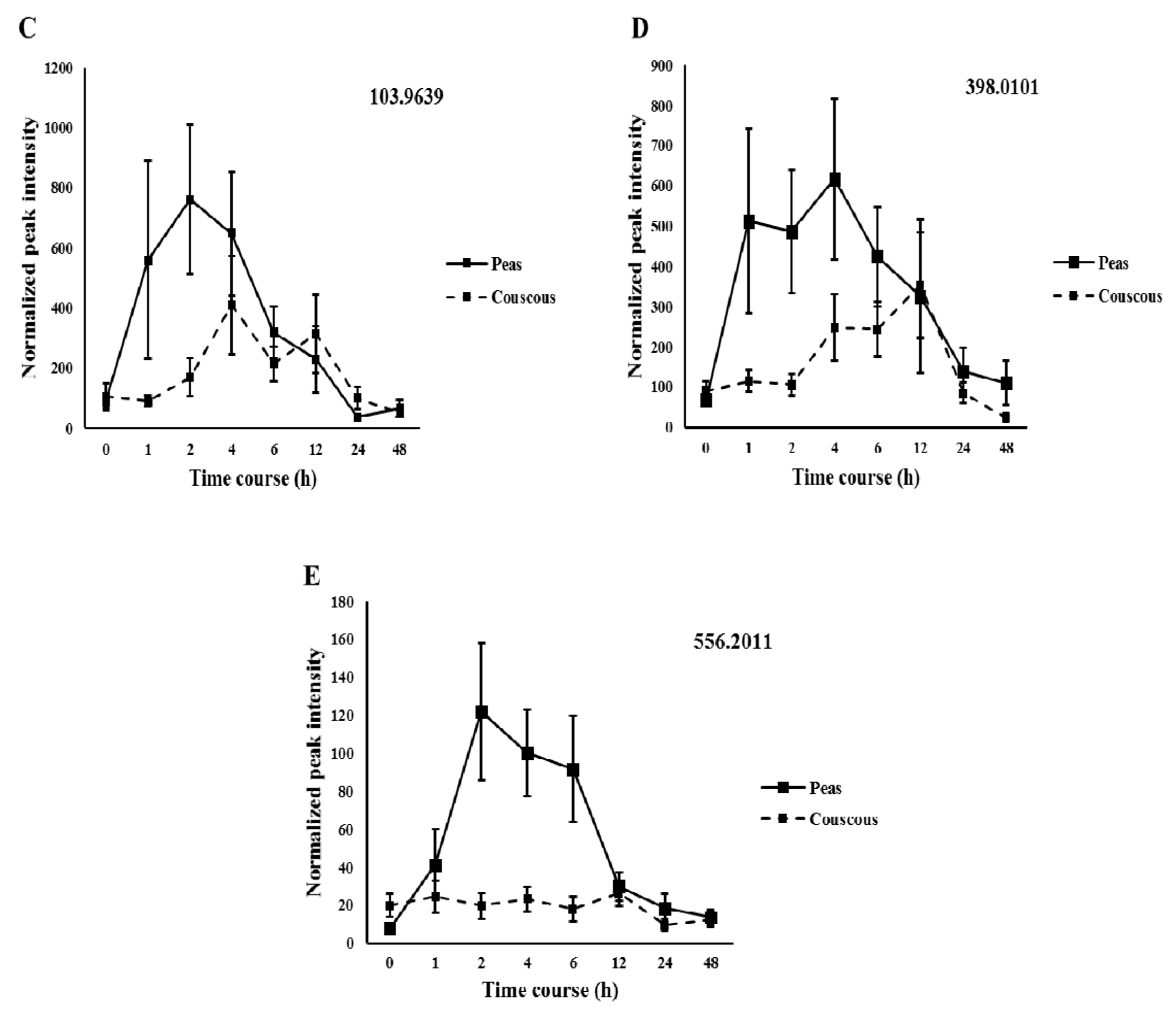
Kinetics of the selected discriminating negative ion mode features obtained from the discovery study represented with accurate mass (176.06838 (**A**); 231.1221 (**B**); 103.9369 (**C**); 398.0101 (**D**); and 556.2011 (**E**)) after pea intake and compared with control food couscous (*p* < 0.05). Values are mean ± SEMs. X-axis values represent time course in hours and Y-axis values represent osmolality normalized peak intensity.

**Figure 3:**
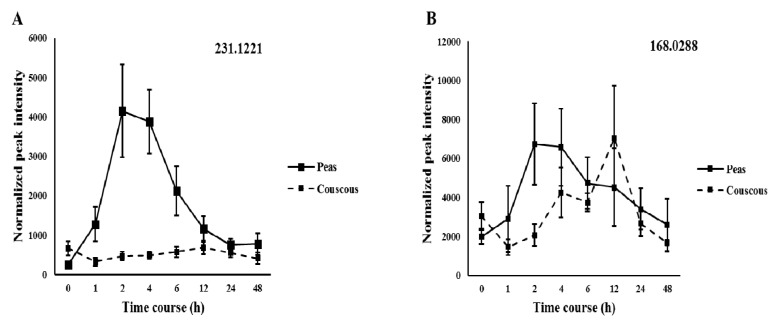

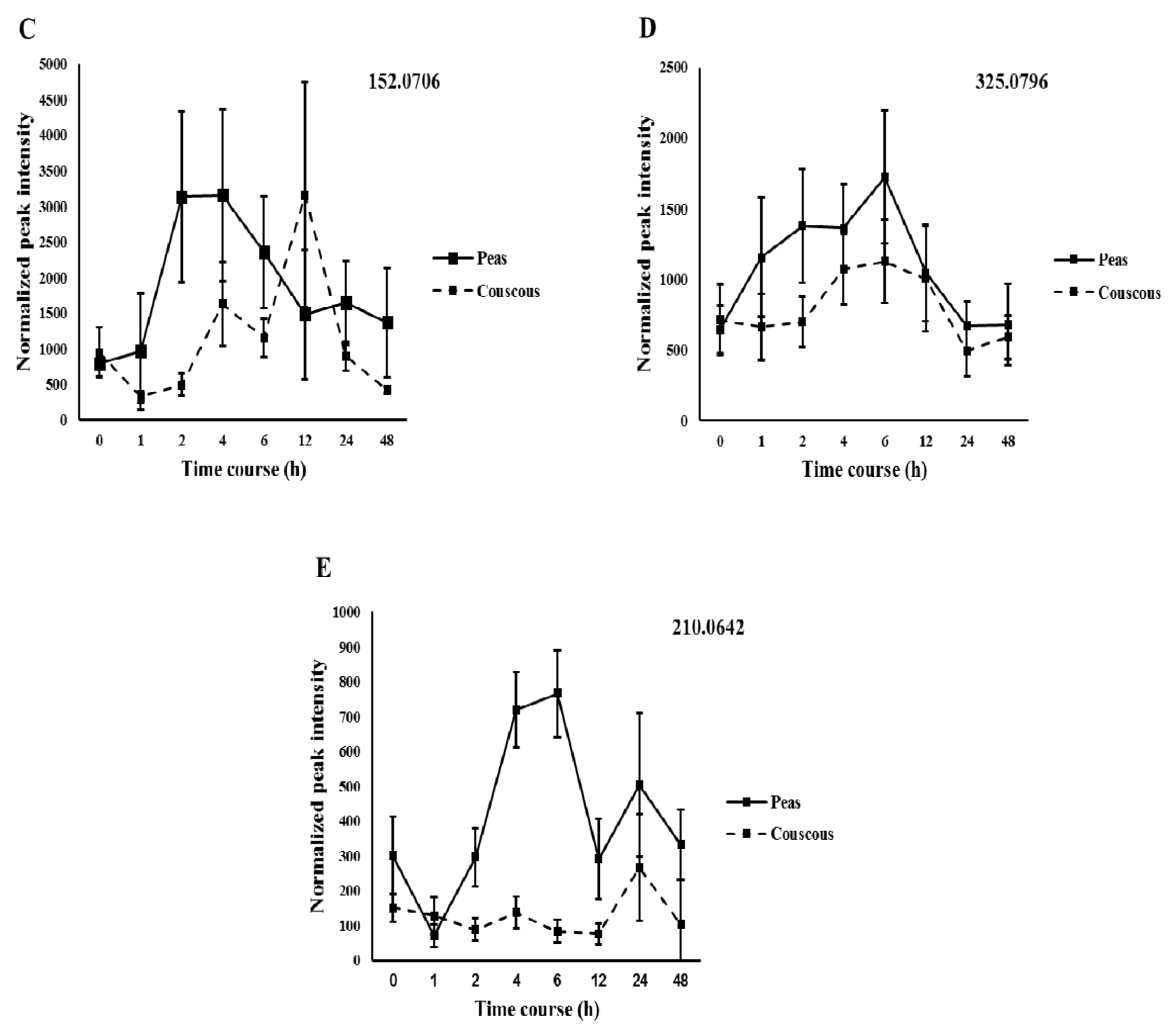
Kinetics of the selected discriminating positive ion mode features obtained from the discovery study represented with accurate mass (231.1221 (**A**); 168.0288 (**B**); 152.0706 (**C**); 325.0796 (**D**); and 210.0642 (**E**)) after pea intake and compared with control food couscous (*p* < 0.05). Values are mean ± SEMs. X-axis values represent time course in hours and Y-axis values represent osmolality normalized peak intensity.

The model was further validated by permutation testing (Q^2^ = (0.0, −0.00618). A variable importance of projection (VIP) plot was generated from the PLS-DA models and the most discriminant features identified (Figure S3). Furthermore, univariate analysis identified features with significant changes over time (Tables S1 and S2). Examination of the interesting features kinetic profiles in comparison to the control food revealed that 10 features (five in negative mode and five in positive mode) displayed a differential time course (Figure 2 and Figure 3). The peak intensity values are represented as mean ± Standard error of the mean (SEM). 

### 3.3. Identification of Potential Biomarkers

To commence identification of the selected features, compound identification was performed using the METLIN database. Using single MS accurate mass data for the molecular ion and its isotopes, a molecular formula for each feature was generated (Table 2).

Table 3 shows a summary of the mass spectral data obtained from MS/MS supporting biomarker annotations.

**Figure 4:**
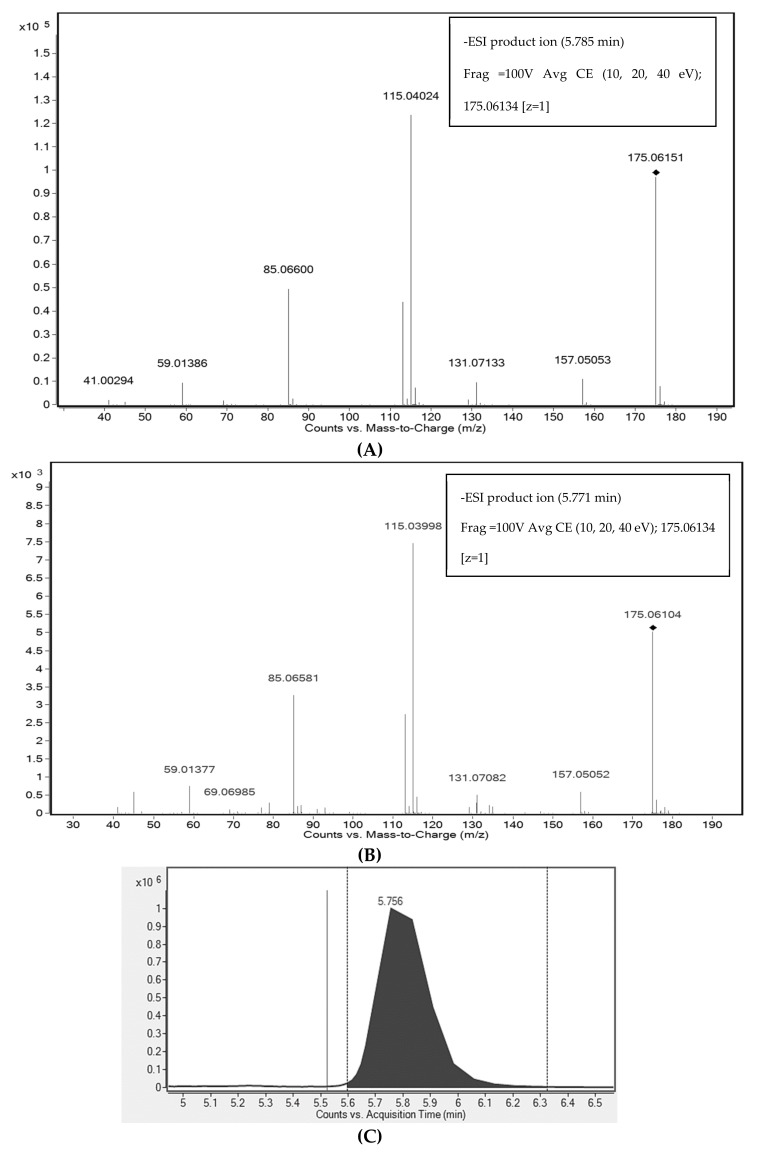

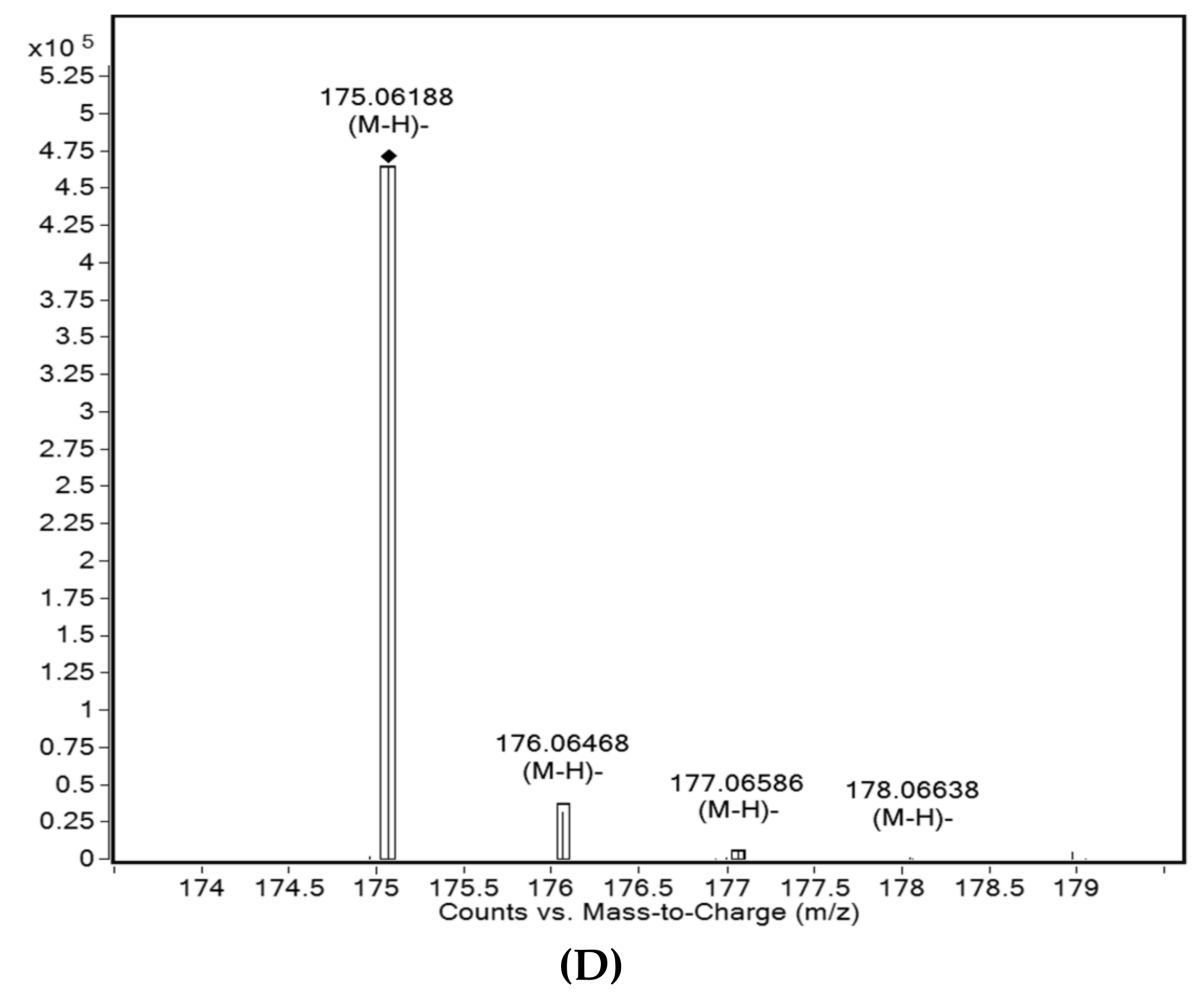
MS/MS spectra of 2-Isopropylmalic acid at average collision energies (10, 20, 40 eV) of: a pure analytical standard (**A**); and in the urine sample (**B**); extracted ion chromatogram (EIC) (**C**); and Find by Formula spectrum (**D**).

2-Isopropylmalic acid (2-IPMA) with *m/z* 175.06134 appeared as a marker compound in negative ionization mode. It was identified based on MS/MS fragmentation match in CFM-ID and Metlin and further verified by authentic analytical standard (Figure 4). The fragments obtained from the standard, 157.05053, 115.04024, 85.0660, and 59.01386, matched with those of the sample in terms of percentage of intensity as well as the fragmentation pattern. The compound was also confirmed by comparison with predicted LC-MS/MS spectra by CFM-ID obtained at 10, 20 and 40 eV (HMDB ID: HMDB00402). The identification is also supported by extract ion chromatogram (EIC), which shows the same retention time and matched isotopic patterns of standard and sample; assessment by find by formula spectrum further supports the compound identification. The excretion kinetics of 2-IPMA displayed a peak excretion at 4 h and exhibited a differential time course when compared with the test food couscous (Figure 2) (*p* < 0.05). 

Asparaginyl valine (Asp-Val) with *m*/*z* 230.1194 was identified as a putative biomarker. The identification was based on the input MS/MS spectra scored against candidate spectra generated and calculated from the HMDB database using CFM-ID. The major fragments *m/z* 230.1145, 213.08785, and 187.1086 matched well between the candidate spectra and the input spectra at low collision energy (10V). For compound identification using CFM-ID, when matching spectra were observed between the candidate and the input spectra, the possible candidates were ranked according to how well their predicted low, medium and high spectra matched the spectra of the test molecule. Jaccard scoring function coefficient was a statistic used for comparing the similarity and diversity of sample sets. However, a range/limit of score cannot be determined as the match patterns can be unique for each identified compound. For Asp-Val, a score of 1.014 was obtained from the spectral match, enabling the identification of the compound [42]. These results were also complemented by high correlation scores obtained by molecular structure correlator utilizing METLIN database. Based on the spectral similarity with commercial spectral libraries, a Level II identification of Asp-Val was proposed. The excretion kinetics of Asp-Val displayed a peak excretion at 4 h and exhibited a differential time course when compared with test food couscous (Figure 2) (*p* < 0.05).

N-Carbamoyl-2-amino-2-(4-hydroxyphenyl) acetic acid (NC) with *m/z* 211.0714 was identified as a putative biomarker. The major fragments with *m/z* 211.07123 and 165.06570 matched between the candidate spectra and the input spectra in CFM-ID. Although the score is on the lower side (0.352857), the database identified the compound to be NC and hence a Level II identification was assigned. Molecular structure correlator also aided in identification of this compound using the METLIN database. The excretion kinetics of NC displayed a peak excretion at 6 h and exhibited a differential time course when compared with test food couscous (Figure 2) (*p* < 0.05).

### 3.4. Demonstration of A Dose–Response for the Key Biomarkers

**Figure 5:**
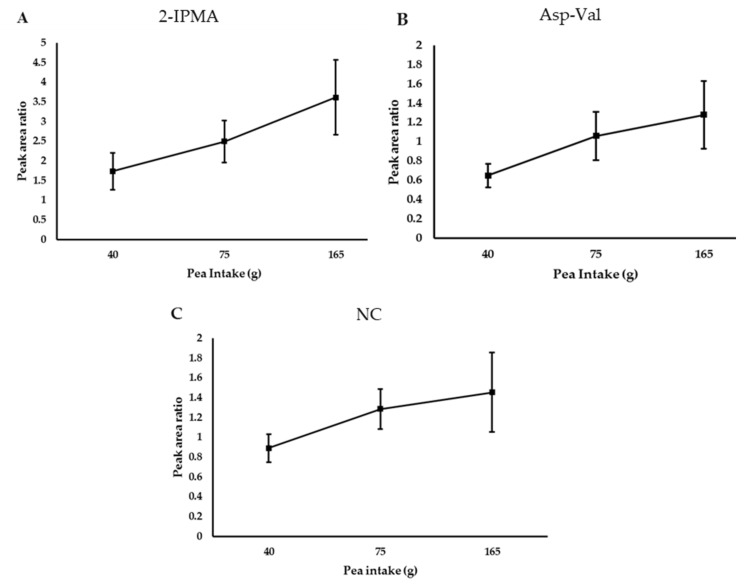
Urinary 2-Isopropylmalic acid (2-IPMA) (**A**); Asparaginyl valine (Asp-Val) (**B**); and N-Carbamoyl-2-amino-2-(4-hydroxyphenyl) acetic acid (NC) (**C**) demonstrated a dose–response to intake of peas from the dose–response study. Values are means ± SEMs (*n* = 12). Participants consumed a low (40 g) (L), medium (75 g) or high (165 g) portion of peas as part of their habitual diet for four consecutive days for each intervention week and first void urine sample collected on the fifth day was analysed. X-axis values represent pea intake (g); Y-axis values represent peak area ratio. Peak area ratio is obtained by dividing the peak area of the sample with that of the internal standard.

Individuals in the dose–response study (*n* = 12) consumed increasing amounts of peas during three weeks in the background of their habitual diet. The characteristics of the participants are described in Table S3 (five males and seven females). The average age was 26 ± 4.9, while the average BMI was 24 ± 2.9 (Table S3). EICs were obtained for the ions of interest (*m/z* 175.06134, 230.1194, 211.0714) and peak area values were determined across low (40 g), medium (75 g) and high (165 g) intake groups. Examination of the data revealed that excretion of 2-IPMA, Asp-Val and NC increased with increasing intake of peas (*p* < 0.05) (Figure 5).

### 3.5. Confirmation of Markers of Pea Intake in An Independent Acute Study

All three metabolites of interest were also confirmed in an independent acute study aimed to find markers of meat intake with pea burger used as a control food. Differential time course profiles were observed for all three metabolites when the control was compared to meat intake (Figure S4). Moreover, the MS/MS fragmentation pattern obtained for the three ions in this independent meal-study also matched well with the fragmentation pattern observed in the pea acute discovery study. 

## 4. Discussion

Identification of objective markers of dietary intake has become an intensive area of research. The current study identified a series of urinary markers specific for pea intake. Importantly, the markers were validated in a dose–response study where participants were consuming their habitual diets, indicating both a dose–response relationship and the robustness of the markers. Further confirmation was provided through measurement in an independent intervention study where pea served as the control food. 

In the present study, we used a multi-pronged approach to identify and validate the biomarkers. Detailed assessment of the biomarkers is of paramount importance to ensure their usefulness both as measures of compliance in intervention studies but also to study diet–disease relationships in epidemiology studies. Recently, several candidate biomarkers across a range of foods have been reported as biomarkers of food intake [43,44]. However, sufficient validity of these markers is lacking to be useful for various applications in nutritional epidemiology. In this context, examination of dose–response has become an essential prerequisite to demonstrate the use of biomarkers in dietary assessment. For example, the discovery and validation of proline betaine as a biomarker of citrus fruit intake has been widely investigated across a number of studies [13,14,15,45,46,47]. In particular, one study identified urinary proline betaine as a biomarker displaying dose–response relationship in a controlled intervention study where participants consumed orange juice at different quantities over a three-week period [21]. Subsequently, calibration curves were developed using proline betaine concentrations and orange juice intake and these calibration curves were used to determine citrus fruit intake in an independent cohort. Overall, the literature clearly establishes the status of proline betaine as a quantifiable biomarker to estimate citrus intake. Similarly, a controlled intervention trial aimed to discover biomarkers of grape intake identified urinary tartaric acid displaying a dose–response relationship with 0, 50, 100, and 150 g/day of reported intake. Calibration curves were developed for urinary tartaric acid against red grape intake (g/day) with a correlation of 0.9 reported between actual and estimated intake projecting a high level of agreement [20]. The combination of both studies clearly demonstrates that well-defined urinary markers can be used to estimate dietary intake. Our present study allows us to add to the literature by demonstrating a dose–response for pea intake. Importantly, the peas were consumed as part of mixed meals constituting the participants’ habitual dietary intake. Future work will examine if the dose–response curves could be used as calibration curves to determine pea intake in a free living population. 

An important aspect of the validation of biomarkers is the biological plausibility of such markers; we argue here that all three metabolites associated with pea intake have plausible biological interpretations [48]. 2-IPMA has been reported to be present in common peas (Alberta Food Composition Database (AFCDB), ID: FDB030115). Our literature search revealed that 2-IPMA is also present in other foods such as melon extracts, tomato and extracts from green leaf lettuce cultivars such as baby, iceberg and romaine [49,50,51]. However, the fact that we observed a dose–response in the background of a habitual diet would suggest that pea consumption is contributing to the majority of urinary 2-IPMA. To our knowledge, there are no reports of studies showing urinary excretion of 2-IPMA reflecting dietary intake. Asp-Val, the Level II identified compound, has been reported to be a breakdown product of protein catabolism or protein digestion (HMDB0028744) [52] and we hypothesise that it is reflective of pea protein consumption. NC belongs to a class of compounds known as N-Carbomoyl-alpha amino acids and is reported to be present in pulses (HMDB0031813). Subsequent confirmation and validation of 2-IPMA, Asp-Val and NC in an independent study comparing with meat protein sources and a dose–response study further evidenced utilization of these metabolites as biomarkers of pea intake. 

There are a number of strengths associated with the present study. A particular strength was the evaluation of a dose–response relationship in the background of a habitual diet confirming the suitability of these markers across a range of intakes. In addition, another unique strength is confirmation of these putative biomarkers in a separate independent intervention study. However, a limitation of the current study is the participants were restricted to European population groups and hence further validation is required in different ethnic populations. Furthermore, the determination of pea intake in an independent cross-sectional cohort could also pave the way for these markers to become objective measures of intake. 

## 5. Conclusions

In summary, the present study demonstrates a successful application of an untargeted metabolomics approach to discover biomarkers of pea intake. 2-IPMA, Asp-Val and NC were discovered as potential biomarkers of pea intake. Key biomarker validation criteria were satisfied with the results obtained from the dose–response study and confirmation of biomarkers in the independent intervention study. The use of such validated biomarkers of pea intake opens up the possibility of objective measures of legume intake. Future work will look into the possibilities of quantifying the biomarkers which could allow application in nutritional epidemiology and to use them as biomarkers of compliance to certain dietary regimes. Combining these biomarkers into a broader panel capturing vegetable intake could open up possibilities for application to objectively assess vegetable intake. 

## Figures and Tables

**Table 1:** Acute study population characteristics in the Intervention study.

Characteristics	Male	Female
No. of participants	6	5
Age, year	26 ± 3	24 ± 5
Height, m	1.8 ± 0.03	1.6 ± 0.08
Weight, kg	77.2 ± 8.06	63.2 ± 8.69
BMI, kg/m^2^	24.1 ± 2.43	24.3 ± 3.7
Waist circumference, cm	82 ± 3.13	74.2 ± 5.84
Hip circumference, cm	100.3 ± 4.63	101.8 ± 6.22
Waist-hip-ratio	0.82 ± 0.03	0.73 ± 0.02

All values are mean ± SD.

**Table 2:** Single MS accurate mass data generated using METLIN database.

RT (min)	Elemental Composition	Type of Molecular Ion	Predicted *m/z*	Observed *m/z*	Error (ppm)
5.78	C_7_H_12_O_5_	[M-H]^−^	175.06134	175.0612	−0.8
1.76	C_9_H_17_N_3_O_4_	[M-H]^−^	230.11463	230.11427	1.58
0.88	C_14_H_12_N_3_O_7_S_2_	[M-H]^−^	397.00439	397.00434	0.13
1.61	C_21_H_36_N_10_S_4_	[M-H]^−^	555.1934	555.192	2.61
0.53		[M-H]^−^		102.9616	
1.8	C_9_H_17_N_3_O_4_	[M+H]^+^	232.12918	232.12942	−1.01
0.90	C_5_H_4_N_4_O_3_	[M+H]^+^	169.03562	169.03631	−4.09
0.8	C_8_H_10_NO_2_	[M+H]^+^	153.07843	153.07826	1.08
1.65	C_14_H_15_NO_8_	[M+H]^+^	326.08704	326.08713	−0.13
3.40	C_9_H_10_N_2_O_4_	[M+H]^+^	211.0767	211.0775	−3.79

RT is the retention time of the given compound in minutes. Predicted *m/z* is the mass to charge ratio of the isotope calculated from the proposed molecular formula. Observed *m/z* is the theoretically observed mass to charge ratio.

**Table 3:** Summary of mass spectral data supporting biomarker annotation.

RT min	Experimental *m/z*	Suggested Ion	Elemental Composition	MS/MS Fragment Ions (Relative Abundancy) ^a,b^	Identification/Annotation ^c^
5.8	175.0611	[M-H]^−^	C_7_H_12_O_5_	115.0399 (100); 175.061 (98.49); 113.0605 (35.27); 85.06581 (31.74)	2-Isopropylmalic acid ^I^
1.8	230.11461	[M-H]^−^	C_9_H_17_N_3_O_4_	187.1087 (100); 230.1145 (46.09); 231.0879 (43.2); 145.0983 (26.56)	Asparaginyl valine ^II^
3.4	211.0727	[M+H]^+^	C_9_H_10_N_2_O_4_	211.072 (100); 165.0663 (43.45); 185.1035 (29.81)	N-Carbamoyl-2-amino-2-(4-hydroxyphenyl)acetic acid ^II^

^a^ Per cent of relative intensity; ^b^ Data obtained at 10 eV collision energy. ^c^ Metabolite Identification (MI) levels as reported according to Metabolomics standards initiative (MSI). ^I^ Compounds identified using standards (Level I); ^II^ Putatively annotated compounds (Level II).

## Supplementary Materials

The following are available online at http://www.mdpi.com/2072-6643/10/12/1911/s1, Figure S1: Flow chart of the recruitment of the participants of the discovery study, Figure S2: Principal component analysis (PCA) of UPLC-QTOF/MS urine data of time point 0 (■) compared with time point 4 h post consumption (□). Q^2^, 0.217; R^2^X, 0.579; t [1], PLS component 1; t [2], PLS component 2, Figure S3: Variable importance of projection (VIP) score plot (≥ 1.5) of 0 h vs. 4 h model (generated from PLS-DA) indicating the most discriminant metabolites in decreasing order. *X*-axis represents the VIP score and *Y*-axis indicates features represented with retention time (RT) and mass. The plot was generated from the data obtained from the discovery study, Figure S4: 2-IPMA (A); ASP-Val (B); and NC (C) metabolites showing a differential time-course when pea burger (control) was compared with beef, chicken and pork in an independent study, Table S1: Significant features identified by one-way repeated measures ANOVA in Negative ion mode, Table S2: Significant features identified by one-way repeated measures ANOVA in Positive ion mode, Table S3: Dose–response study population characteristics.
