# Supplementary material for: A Metabolomics Approach to the Identification of Urinary Biomarkers of Pea Intake
Source: Nutrients. 2018 Dec 4;10(12):1911. doi: 10.3390/nu10121911 (PMC6315433; doi:10.3390/nu10121911)
Supplement: Supplementary file 1 [file nutrients-10-01911-s001.pdf]

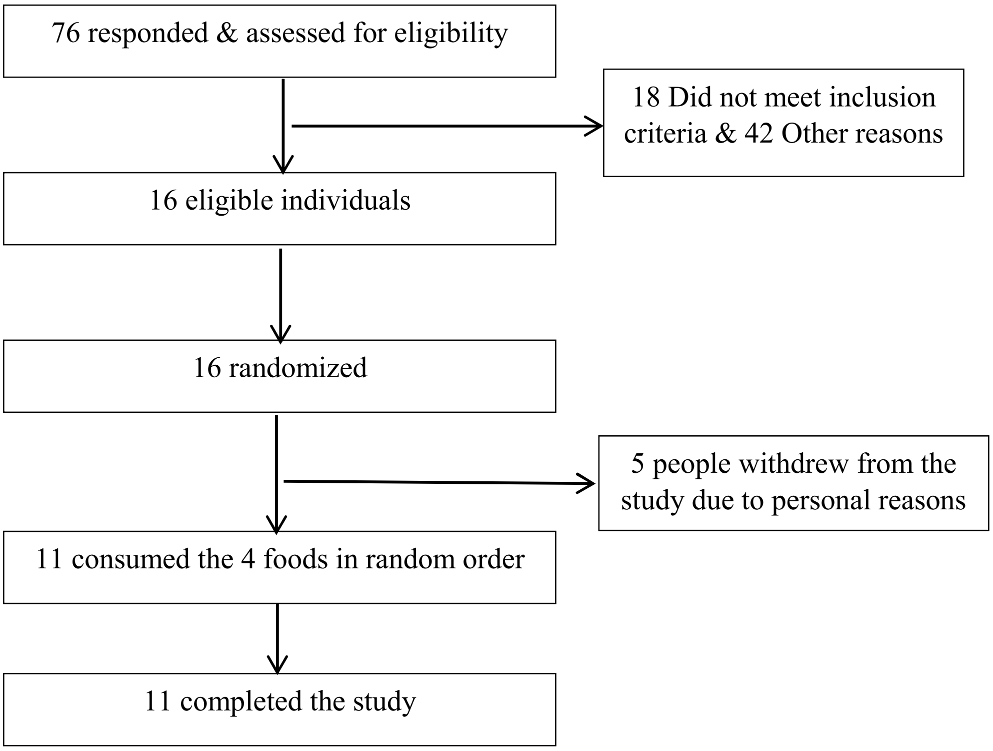

Figure S1. Flow chart of the recruitment of the participants of the discovery study.

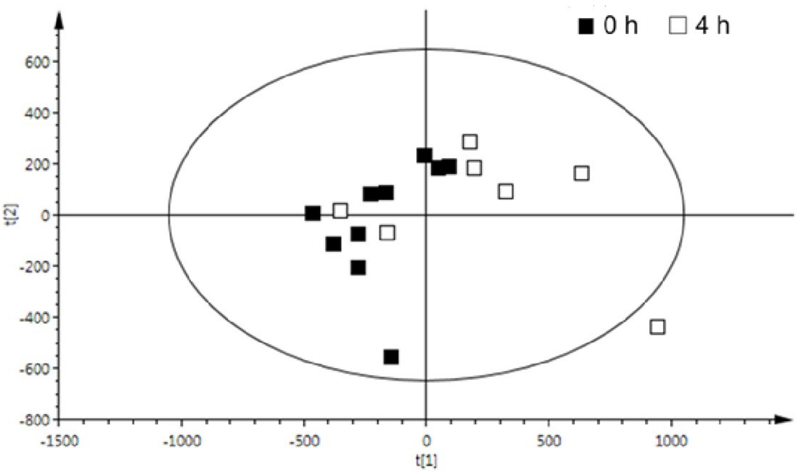

Figure S2. Principal component analysis (PCA) of UPLC-QTOF/MS urine data of time point 0 (■) compared with time point 4 hours post consumption (□). Q^2, 0.217; R^2X, 0.579; t [1], PLS component 1; t [2], PLS component 2.

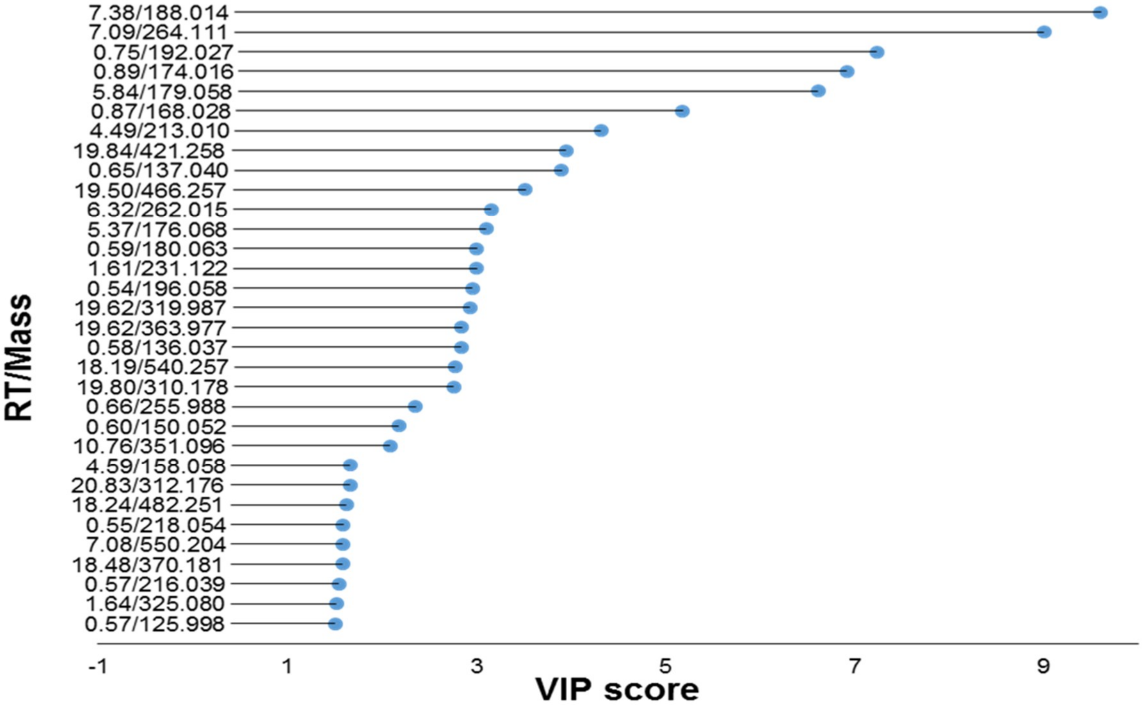

Figure S3. Variable importance of projection (VIP) score plot (≥ 1.5) of 0 h vs. 4 h model (generated from PLS-DA) indicating the most discriminant metabolites in decreasing order. *X*-axis represents the VIP score and *Y*-axis indicates features represented with retention time (RT) and mass. The plot was generated from the data obtained from the discovery study.

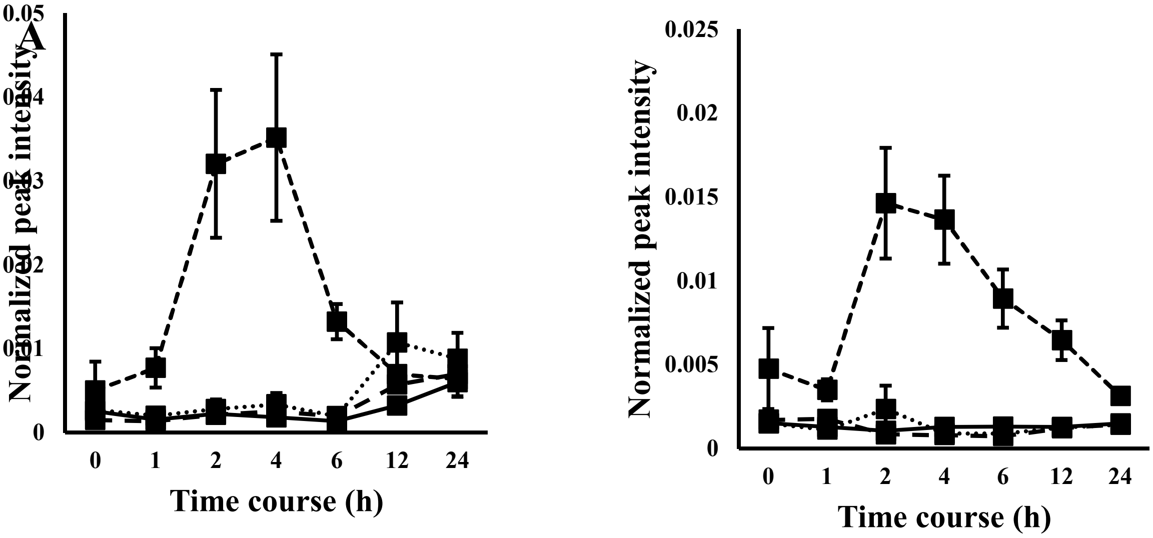

(A) (B)

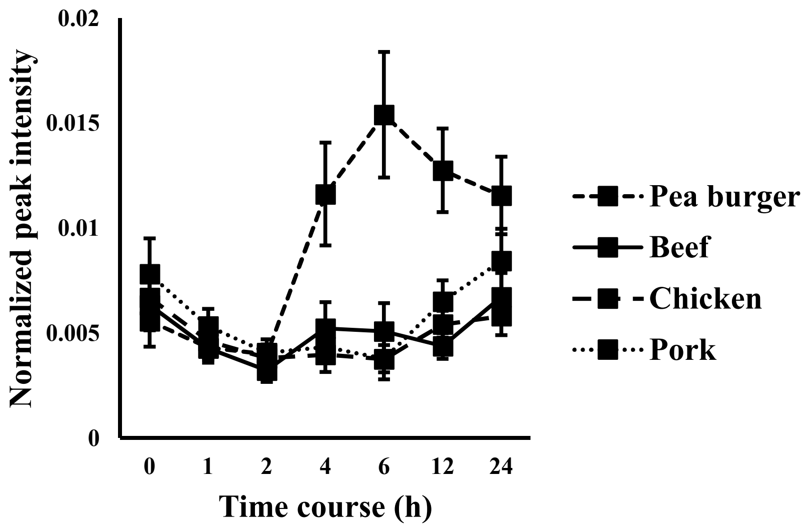

(C)

Figure S4. 2-IPMA (**A**), ASP-Val (**B**), NC (**C**) metabolites showing a differential time-course when pea burger (control) was compared with beef, chicken and pork in an independent study.

Table S1. Significant features identified by One-way repeated measures ANOVA in Negative ion mode.

Feature [a]	Average Normalized Intensity [b]	*p*-Value [c]
1.051/193.9884	253.49 ± 244.46	1.7781E-05
0.989/362.017	74.85 ± 66.14	5.1499E-05
0.953/223.9991	214.61 ± 193.96	5.1499E-05
18.01/434.2151	279.62 ± 361.55	5.1499E-05
16.012/200.105	435.69 ± 354.86	0.00011
9.168/274.0148	1089.83 ± 843.33	0.00015
18.445/226.1203	163.51 ± 75.13	0.00023
0.927/392.0272	84.66 ± 80.30	0.00065
13.442/266.1264	99.74 ± 120.02	0.00083
13.098/190.1203	22.80 ± 17.42	0.00083
1.358/320.0742	275.60 ± 295.97	0.00083
13.643/380.1682	255.94 ± 239.62	0.00083
16.229/541.2194	18.64 ± 12.69	0.00083
17.899/569.2504	17.32 ± 11.43	0.00093
17.68/478.2404	61.09 ± 48.18	0.00135
8.806/304.0252	93.90 ± 53.13	0.00135
18.97/436.2306	193.31 ± 174.40	0.00135
18.33/320.1472	174.06 ± 184.65	0.00255
1.611/556.2011	53.10 ± 44.58	0.00373
0.577/79.9258	21.72 ± 19.45	0.00394
14.885/188.1049	487.03 ± 327.43	0.01105
16.086/406.184	482.69 ± 492.76	0.01139
19.233/426.1251	42.59 ± 18.81	0.02400
19.605/449.3133	188.18 ± 100.20	0.02686
0.557/103.9639	338.04 ± 283.74	0.02747
0.643/214.0234	243.25 ± 137.86	0.03447
19.633/447.2982	351.25 ± 207.51	0.03489
0.855/493.9674	23.49 ± 13.73	0.03876

16.127/246.1465	129.97 ± 98.87	0.04243
5.78/176.0684	993.78 ± 997.29	0.04243
0.879/130.0266	5223.42 ± 1462.60	0.04243
19.08/290.1288	62.47 ± 22.90	0.04243
0.568/188.0443	736.90 ± 399.13	0.04520
19.427/584.3185	125.35 ± 76.55	0.04520
11.879/412.1148	60.51 ± 24.65	0.04520
1.768/231.1218	786.78 ± 645.81	0.04520
0.891/174.0164	18397.71 ± 3839.19	0.04520
19.788/ 513.276	856.84 ± 518.44	0.04520
17.452/224.1047	186.48 ± 86.47	0.04520
6.665/189.0425	353.07 ± 128.96	0.04703
18.764/506.2721	19.49 ± 8.81	0.04721

[a] Feature is represented as retention time (RT) in min/ Mass [b] Intensities averaged across time-points for all the samples and normalized to osmolality [c] p-value obtained from multiple testing correction by false discovery rate

Table S2. Significant features identified by One-way repeated measures ANOVA in Positive ion mode.

Features [a]	Average Normalized Intensity [b]	*p*-Value [c]
18.044/451.241	45.66 ± 37.99	0.00023
0.879/214.0965	166.77 ± 106.64	0.00386
18.632/553.2159	123.21 ± 134.44	0.00524
1.624/231.1218	1795.11 ± 1470.13	0.00550
19.592/477.2415	105.74 ± 42.14	0.00550
8.205/492.2583	22.22 ± 10.57	0.00841
1.335/320.0737	126.11 ± 120.71	0.00891
18.613/513.3401	40.60 ± 26.91	0.01205
18.625/558.1722	50.32 ± 54.35	0.01596
3.476/210.0642	409.78 ± 236.09	0.02110
2.133/93.0212	72.73 ± 22.22	0.02229
19.612/447.2977	1227.72 ± 836.43	0.02357
1.467/289.1271	546.80 ± 190.10	0.02841
10.479/329.184	860.62 ± 297.27	0.03080
19.371/555.3867	41.57 ± 24.61	0.03464
18.35/355.2357	602.21 ± 250.16	0.03523
17.71/649.3672	15.52 ± 10.40	0.03523
0.855/229.9835	589.57 ± 654.53	0.03523
19.164/623.3313	34.20 ± 17.09	0.03626
19.601/472.2667	35.73 ± 24.95	0.03877
14.889/188.1045	275.54 ± 250.86	0.04175
1.029/379.0311	34.88 ± 17.17	0.04812

[a] Feature is represented as retention time (RT) in min/ Mass [b] Intensities averaged across time-points for all the samples and normalized to osmolality [c] *p*-value obtained from multiple testing correction by false discovery rate

Table S3. Dose response study population characteristics.

Characteristics	Male	Female
No. of participants	5	7
Age, year	26 ± 3.5	26 ± 6.02
Height, m	1.8 ± 0.07	1.6 ± 0.05
Weight, kg	79.3 ± 10.23	62.2 ± 9.42

BMI, Kg/m^2	24.9 ± 2.26	23.5 ± 3.31
Waist circumference, cm	82.8 ± 6.42	74 ± 8.07
Hip circumference, cm	102.7 ± 7.26	99.6 ± 7.21
Waist-hip-ratio	0.81 ± 0.02	0.74 ± 0.04

All values are mean ± SD.
